# Associations of Weather Patterns and Shelter Availability With Healthcare Utilization Among People Experiencing Homelessness in a Rural Region

**DOI:** 10.51894/001c.144123

**Published:** 2025-09-30

**Authors:** Zi Jie Wei

**Affiliations:** 1 College of Osteopathic Medicine Michigan State University

## 39

### INTRODUCTION/BACKGROUND

Little is known about healthcare utilization patterns among people experiencing homelessness (PEH) in rural areas, who often face unique healthcare access barriers related to geographic isolation and resource shortages. It is also unclear how weather patterns and shelter availability impact healthcare utilization among PEH.

### OBJECTIVES/HYPOTHESIS

We described trends in emergency department (ED) utilization and acute hospitalizations among PEH in a rural region of Northwest Michigan and examined whether weather patterns and the availability of shelters for PEH were associated with healthcare utilization.

### METHODS

This retrospective study included data on 1,524 ED visits and 301 acute hospitalizations for PEH at Munson Medical Center in rural Northwest Michigan (January 1, 2018–October 10, 2022). PEH were identified by shelter addresses in electronic health records. Healthcare visits were categorized by type using ICD-10 codes. Data on temperature, precipitation, and snow depth were obtained from the National Weather Service. Descriptive statistics assessed healthcare utilization trends. Pearson’s Chi-squared tests examined differences in diagnostic categories by gender and age group (<45 vs. >45 years). Linear regression models evaluated associations between weather and healthcare utilization.

### RESULTS

Mental/behavioral disorders accounted for 66.3% of ED visits and 84.4% of hospitalizations among PEH. Females had a higher prevalence of ED visits for endocrine/metabolic and respiratory diseases, while circulatory system diseases, injuries, and pain in chest/throat were more common among males (all p<0.01). Most diagnostic categories were more common among PEH >45 years, except for infections (p=0.02) and pregnancy-related disorders (p=0.002). Healthcare utilization fluctuated over time but was not linked to shelter availability. Higher maximum daily temperature was positively associated with ED visits (β=0.44, 95% CI=0.21, 0.67), while total precipitation was associated with fewer ED visits (β=-1.43, 95% CI=-2.5, -0.33). Greater cumulative snow depth was linked to increased ED visits (β=0.56, 95% CI=0.09, 1.0) and hospitalizations (β=0.18, 95% CI=0.03, 0.32).

### DISCUSSION/CONCLUSIONS

Like urban settings, healthcare utilization among PEH in rural Northwest Michigan is predominantly related to mental and behavioral health. Our findings suggest weather-related impacts on ED visits and hospitalizations among PEH in rural regions. Providing accessible healthcare services during weather events may help mitigate ED use.

